# A Case of Pancreatic Fistula Following Left Nephrectomy: A Rare Complication

**DOI:** 10.7759/cureus.81157

**Published:** 2025-03-25

**Authors:** Fatima I Hsayan, Amani El Abed, Sondos Naous, Ahmad H Alhajj Mohammad, Antoine S Geagea

**Affiliations:** 1 Gastroenterology, Lebanese University Faculty of Medicine, Beirut, LBN; 2 Internal Medicine, Lebanese University Faculty of Medicine, Beirut, LBN; 3 Gastroenterology and Hepatology, Lebanese Hospital Geitaoui University Medical Center, Beirut, LBN

**Keywords:** drain, laparotomy, nephrectomy, pancreatic, pancreatic fistula

## Abstract

Pancreatic fistula is a rare but serious complication that may arise following surgery, particularly in oncological procedures. Intraoperative pancreatic injury is an infrequent complication associated with left nephrectomy. We present the case of a 77-year-old male patient who underwent a left nephrectomy for a rare renal pelvis tumor, specifically sarcomatoid urothelial carcinoma. After two consecutive laparotomies, the patient developed a pancreatic fistula, as evidenced by excessive left drain output and elevated amylase levels in the collected fluid. The fistula was managed effectively with subcutaneous somatostatin analog (Sandostatin) injections, parenteral nutrition, and a strict nothing by mouth (NPO) regimen.

This case underscores the potential risk of pancreatic fistula following radical nephrectomy, highlighting the importance of early diagnosis and appropriate management strategies. Unfortunately, the patient's recovery was complicated by intra-abdominal bleeding and septic shock, which ultimately led to his death.

## Introduction

A pancreatic fistula, defined as any leakage of enzyme-rich fluid from the pancreas, can occur after surgery, acute pancreatitis, or trauma [1]. Intraoperative pancreatic injury is a relatively uncommon complication following left radical nephrectomy, occurring in about 2.1% of cases [2].

Due to the intimate anatomical relationship between the pancreas and the left kidney, surgical trauma to the pancreas during nephrectomy is an infrequent but recognized complication [3]. During left renal surgery, the distal or tail segment of the pancreas is the most commonly injured part, owing to its anatomical proximity to the surgical field [2,4]. The left kidney and pancreatic tail are separated only by the anterior lamina of the renal fascia [4]. Pancreatic tail injury following left nephrectomy has been reported with an incidence rate of 2.1% [2]. These pancreatic injuries can present as an abscess, fistula, or pseudocyst [3]. Several risk factors are associated with postoperative pancreatic fistula, including male gender, obesity (BMI > 25 kg/m^2^), advanced age, and a C-reactive protein level exceeding 20 mg/dL on postoperative day three [5].

In this article, we present a rare case of a pancreatic fistula following a left nephrectomy for sarcomatoid urothelial carcinoma, which occupied the entire left aspect of the abdominal cavity.

## Case presentation

A 77-year-old male patient, a smoker with no known food or drug allergies, had a medical history of hypertension, dyslipidemia, coronary artery disease, benign prostatic hyperplasia, and chronic obstructive pulmonary disease. He presented to the emergency department with a three-month history of vague, non-specific abdominal pain, abdominal distension, and increased abdominal girth, which he had attributed to weight gain.

Initial laboratory tests revealed normocytic anemia, severe leukocytosis, and acute kidney injury, as shown in Table 1.

**Table 1:** The patient's laboratory test results

Laboratory tests	Results	Reference range
Hemoglobin	10.5 g/dL	14-18 g/dL
Mean corpuscular volume	91.6 fL	80-96 fL
White blood cells	86,340 cells/mm³	4,800-10,800 cells/mm³
Neutrophils	96%	60-70%
C-reactive protein	319 mg/L	0-6 mg/L
Creatinine	3.11 mg/dL	0.61-1.24 mg/dL
Blood urea nitrogen	72 mg/dL	8-25 mg/dL

**Figure 1:**
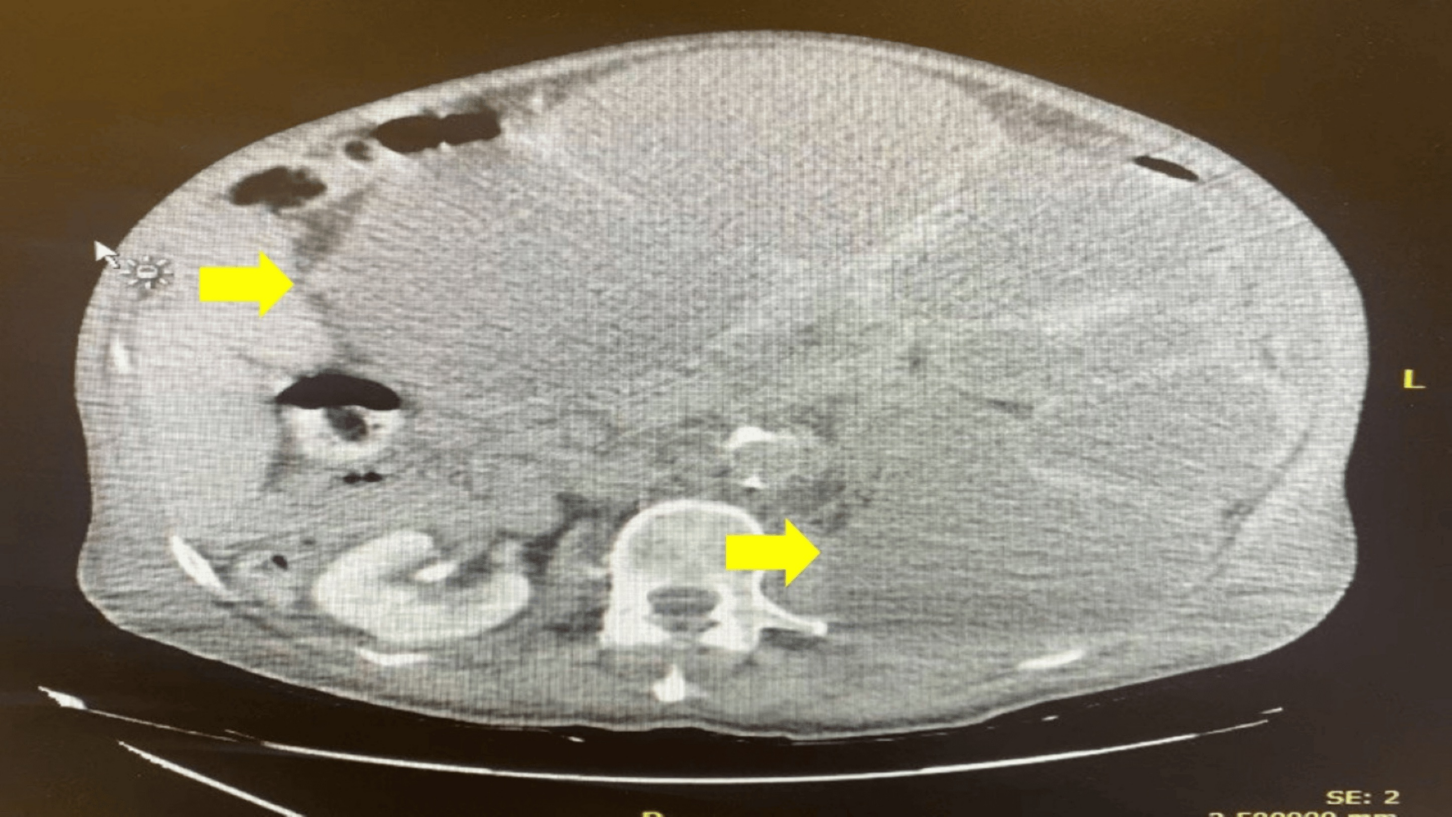
A CT scan of the abdomen and pelvis without IV contrast demonstrated a severely enlarged left kidney occupying the left side of the abdominal cavity. Radiological findings were suggestive of chronic ureteral obstruction complicated by severe xanthogranulomatous pyelonephritis of the left kidney (yellow arrows).

Upon presentation, a CT scan of the abdomen and pelvis without contrast revealed a severely enlarged left kidney occupying the left side of the abdominal cavity. The renal cortex was extremely thinned and replaced by hypodense areas, and a 2.2 cm obstructive left ureteral stone was observed. These findings were suggestive of chronic ureteral obstruction complicated by severe xanthogranulomatous pyelonephritis of the left kidney (Figure 1).

The patient was started on broad-spectrum antibiotics with meropenem and underwent an echo-guided nephrostomy of the left kidney, draining 4.5 liters of dark, black-colored fluid. Four days after admission, the patient underwent laparotomy with left nephrectomy. The left kidney was sent for pathology, which revealed a sarcomatoid urothelial carcinoma of the renal pelvis.

Blood samples for BCR-ABL gene testing and flow cytometry were negative, as shown in Table 2.

**Table 2:** Blood tests for BCR-ABL gene and flow cytometry analysis

Blood tests	Results
BCR-ABL gene	Negative, translocation T(9:22) was not detected
Flow cytometry	Showed a predominance of granulocytes with no distinctly increased or aberrant blast and no phenotypic support for acute leukemia or increased blasts.

**Figure 2:**
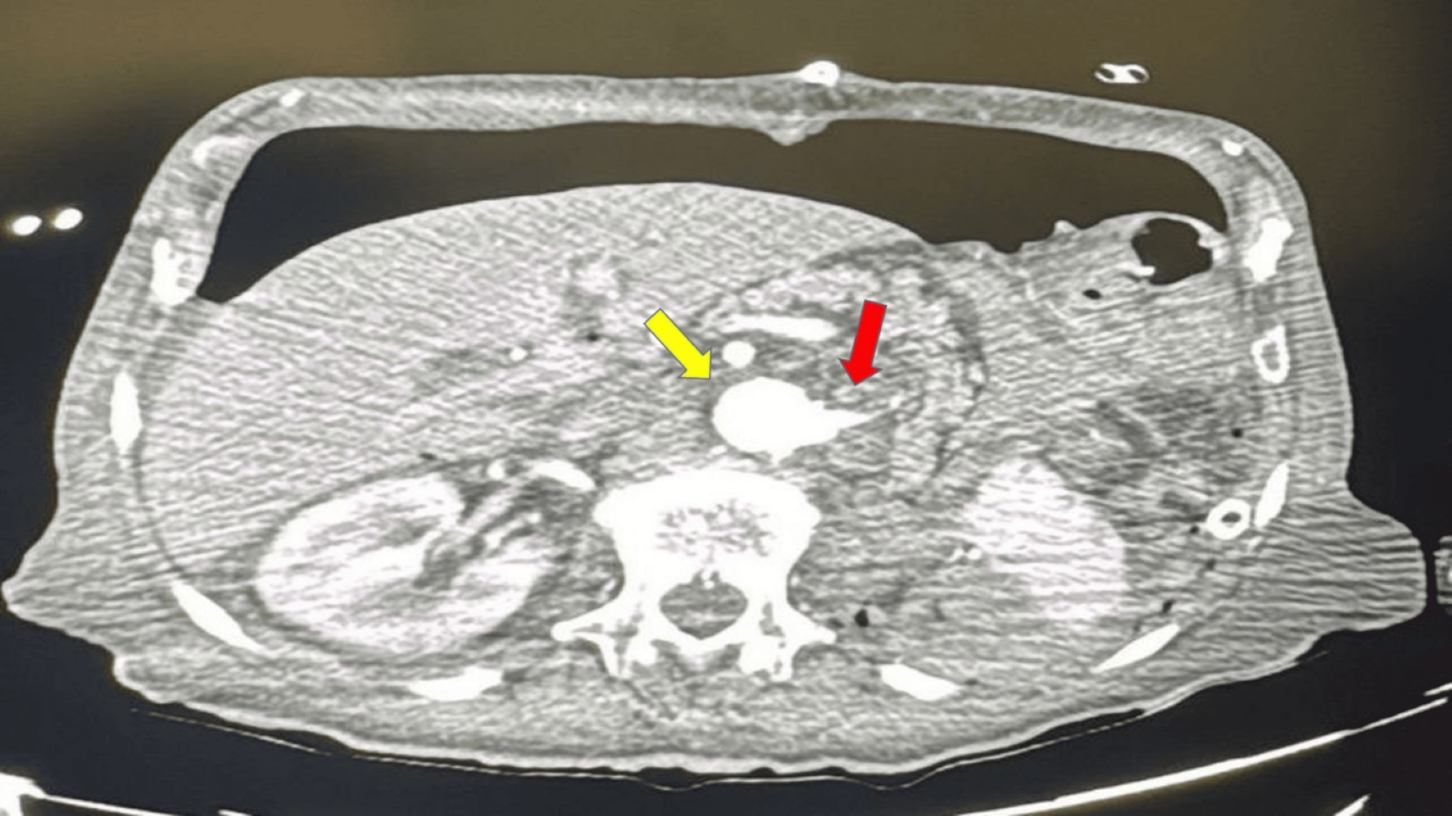
The CT angiography showed active bleeding from the left renal artery (red arrow) arising from the abdominal aorta (yellow arrow).

Three days after nephrectomy, the patient developed hypotension, a decrease in hemoglobin, and significant bloody drainage from the surgical site. An urgent CT angiography of the abdomen revealed active bleeding from the left renal artery, arising from the abdominal aorta (Figure 2).

The bleeding was controlled through an urgent laparotomy and ligation of the bleeding left renal artery, along with multiple sutures to the pancreas following iatrogenic manipulation. At the end of the surgery, two surgical drains (right and left) were placed. Over the next five days, the drained fluid from both drains was serosanguineous, with approximately 200 cc/day drained from each side. Of note is that one week after the second operation, the left drain output increased to about 1,250 cc/24 hours, while the right drain output remained no more than 250 cc/24 hours.

A diagnosis of pancreatic fistula was suspected and confirmed by analysis of the left drain secretion, which showed elevated levels of amylase (8,508 U/L) and lipase (33,643 U/L). In contrast, serum lipase was normal at 56 U/L (reference range: 13-60 U/L), and serum amylase was normal at 79 U/L (reference range: 28-100 U/L). Magnetic resonance cholangiopancreatography (MRCP) was requested but not performed. Subcutaneous Sandostatin injections were initiated, along with parenteral nutrition and no oral intake. It was noted that the drainage from the left drain gradually decreased to 350 cc/24 hours after five days of appropriate management.

Sadly, his hospital course was complicated by respiratory failure, for which he was intubated. After extubation, he developed septic shock secondary to pyelonephritis and subsequently passed away.

## Discussion

In summary, our case represents a rare instance of sarcomatoid urothelial carcinoma of the left renal pelvis with severe enlargement of the left kidney requiring surgery, complicated by renal vessel bleeding, pyelonephritis, and a pancreatic fistula following two consecutive surgeries, respectively.

The most important diagnostic criterion of pancreatic fistula is a three-fold increase in the amylase level in the fluid collected from the fistula [2]. In our case, the drainage fluid amylase measurement was 8,508 U/L. Strong consideration should be given to leaving a drain in the surgical bed after a difficult dissection, as it allows for prompt diagnosis in the postoperative period of an unrecognized pancreatic injury. These drains should remain in place until the drainage is less than 50 mL/24 hours and the fluid amylase has returned to serum levels [4].

Pancreatic fistulas following nephrectomy are rare [6]. The amount of drainage from a pancreatic fistula varies depending on the site of injury. If the main duct located in the tail of the pancreas is injured, it will cause less drainage than a damaged main duct in the head of the pancreas [3]. Notably, the diagnosis of pancreatic injury is rarely made intraoperatively; indeed, 75% of these injuries are diagnosed in the postoperative period [2]. In the majority of cases, pancreatic fistula develops in the early postoperative period and can lead to fatal complications, such as infection and bleeding [6]. Hence, a high level of suspicion of pancreatic injury is required for early recognition after a difficult operation. However, cases of delayed pancreatic fistula have also been reported in the literature [2].

According to general surgical experience, most fistulas close within three weeks and resolve with drainage alone [4]. In 30% to 50% of cases, conservative treatment, which includes discontinuation of oral nutrition, antibiotic therapy based on the identified microorganisms, and somatostatin analog administration to suppress pancreatic excretion function, is successful [2]. Our patient was started on total parenteral nutrition (TPN) and Sandostatin and was kept nothing by mouth (NPO) when a diagnosis of pancreatic fistula was confirmed by analysis of the fluid from the drain. Five days following the initiation of Sandostatin, the amount of fluid drained decreased from 1,250 mL/24 hours to 350 mL/24 hours. However, if the pancreatic fistula shows substantial resistance to conservative treatment, surgery may be required. According to a recent report, operative treatment was more successful than medical therapy in most cases (94% vs. 31%) [6]. When oral feeding is resumed, a low-fat diet should be considered [4].

It is also worth noting that the sarcomatoid variant of urothelial carcinoma (SVUC) is a rare variant of urothelial carcinoma (SVUC) of the renal pelvis, which often extends into the renal parenchyma. SVUC accounts for 0.1% of 0.3% of all urinary tract urothelial carcinoma cases [7, 8]. The first case of renal pelvis urothelial carcinoma was reported by Piscioli et al. in 1984 [6]. An SVUC is a high-grade malignant tumor containing both epithelial and mesenchymal cells, manifesting with aggressive clinical behavior and carrying a poor prognosis [7,8]. Tumors arising from the renal pelvis have the worst prognosis, with a mean survival of less than nine months [7]. This disease has a male-to-female predominance, with a male-to-female ratio of approximately 2:3, and usually manifests in individuals over 50 years of age [8]. These tumors, also known as carcinosarcoma or spindle cell carcinoma, consist microscopically of urothelial, glandular, or small cell components, with varying degrees of differentiation. Carcinoma in situ is found in 30% of these cases [8].

## Conclusions

To conclude, our case presents a rare instance of sarcomatoid urothelial carcinoma of the renal pelvis with a rare post-left nephrectomy complication-pancreatic fistula-successfully managed with Sandostatin injection, an NPO regimen, and TPN. Unfortunately, the hospital course was complicated by intra-abdominal bleeding, respiratory failure, and pyelonephritis, leading to septic shock and ultimately death.
